# Plasma Proteomic Signatures Predict Incident Amyotrophic Lateral Sclerosis: A Prospective Study Stratified by *C9orf72* Expansion

**DOI:** 10.1002/jcsm.70405

**Published:** 2026-10-20

**Authors:** Wei‐Ming Su, Qing‐Qing Duan, Nan Zhang, Sheng‐Yi He, Ru‐Yin Liu, Xiang‐Jin Wen, Xiao‐Jing Gu, Yi Wang, Bei Cao, Yong‐Ping Chen

**Affiliations:** ^1^ Department of Neurology and Institute of Neurology, West China Hospital Sichuan University Chengdu Sichuan China; ^2^ Mental Health Center, West China Hospital Sichuan University Chengdu Sichuan China; ^3^ Department of Pathophysiology, West China College of Basic Medical Sciences and Forensic Medicine Sichuan University Chengdu Sichuan China

**Keywords:** amyotrophic lateral sclerosis, *C9orf72*, NEFL, plasma proteomics, risk prediction

## Abstract

**Background:**

Amyotrophic lateral sclerosis (ALS) is a progressive and fatal neurological disorder for which reliable preclinical biomarkers to predict disease onset are currently lacking. Plasma proteomics offers a promising avenue for early detection. The identification of plasma proteins and pathways associated with incident ALS holds potential to improve early diagnosis, enable risk stratification and provide insights into disease mechanisms.

**Methods:**

Employing the Olink Explore platform, we quantified 2920 plasma proteins within a prospective cohort of UK Biobank participants. Multivariate Cox proportional hazards regression was utilized to identify proteins associated with incident generalized ALS, C9‐ALS (*C9orf72*‐associated ALS) and non‐C9‐ALS. Enrichment analysis was conducted to delineate the biological pathways implicated by the identified proteins. Associations between plasma protein levels and the age at onset (AAO) of ALS were assessed via linear regression, while a multivariate Cox model was employed to evaluate the relationships between protein levels and ALS survival. We subsequently assessed potential causal relationships using Mendelian randomization (MR) and colocalization and analysed protein cross‐sectional temporal patterns in ALS.

**Results:**

In this prospective cohort study, we followed 52 584 participants from the UK Biobank for a mean of 11.8 years. During this period, 291 (0.55%) incident cases of ALS were identified, among whom 34 individuals carried the *C9orf72* hexanucleotide repeat expansion mutation. Nine proteins demonstrated associations with all‐type ALS risk (FDR < 0.05): NEFL (HR: 2.897), CSRP3 (HR: 1.233), MYBPC1 (HR: 1.352), MEGF10 (HR: 1.744), MYOM3 (HR: 1.289) and THBS4 (HR: 1.570) were identified as risk factors, whereas EGFLAM (HR: 0.402), ITGAV (HR: 0.284) and CD248 (HR: 0.573) exhibited protective effects. NEFL (HR: 2.742) and three additional proteins were primarily associated with non‐C9‐ALS, while only NEFL (HR: 4.403) and COL5A1 (HR: 3.072) showed significant associations with C9‐ALS. When combined with age and sex, these proteins yielded area under the curve (AUC) values exceeding 0.750 for predicting ALS at 3, 5 and 10 years. Furthermore, elevated NEFL levels were correlated with an earlier onset of non‐C9‐ALS and began to increase approximately 9.27 years prior to diagnosis, reaching a peak 1–2 years before clinical presentation in non‐C9ALS cases via cross‐sectional temporal analysis. Pathway analysis implicated muscle‐related, extracellular matrix, energy metabolism and inflammatory pathways.

**Conclusions:**

This investigation uncovers extensive alterations in plasma proteins associated with ALS risk, revealing potential novel biomarkers for both sporadic and *C9orf72*‐linked forms of the disease. These results enhance our comprehension of early ALS pathogenesis and provide a foundation for subsequent biomarker identification and the development of targeted therapeutic strategies.

## 1 Background

Amyotrophic lateral sclerosis (ALS) is a fatal and heterogeneous neurodegenerative disease of the central nervous system, characterized by challenges in early diagnosis and an unfavourable prognosis, with a median survival of 3–5 years following symptom onset [1]. Neurodegeneration in ALS commences long before clinical manifestations emerge; by the time symptoms such as limb weakness, dysphagia, dysarthria or respiratory compromise become evident, a significant fraction of motor neurons has already degenerated. The pathogenesis of ALS is multifactorial, arising from an interplay of genetic predisposition and environmental factors [2]. A key genetic determinant involves the hexanucleotide repeat expansion (HRE) in *C9orf72*, which represents the most frequent genetic cause of ALS populations of European ancestry [2]. Nevertheless, the biological pathways disrupted in ALS remain incompletely understood. A major contributing factor is the limited insight into the early pathogenic alterations in ALS, which has resulted from a pathological understanding of the disease that is predominantly derived from observations in typical clinical cases.

Although previous proteomic analyses of plasma, brain, cerebrospinal fluid (CSF) and muscle [3, 4, 5, 6, 7] have revealed several ALS‐associated proteins, the generalizability of these findings remains constrained, and key aspects of the disease pathology warrant further exploration. A key limitation of existing studies [3, 4, 5, 6, 7] is their predominant focus on symptomatic ALS patients, which stems from the challenge of recruiting individuals in the early preclinical stage and results in a gap in our understanding of proteomic features during the asymptomatic period. Moreover, previous proteomic investigations [4, 5, 6, 7] have largely relied on cross‐sectional designs, an approach that is susceptible to reverse causality and may undermine the validity of the reported associations. The general constraint of modest sample sizes in these studies further diminishes the robustness of the findings [6, 7]. Third, some studies [8, 9] have not adequately controlled for the genetic background of ALS. Finally, while the *C9orf72* HRE is strongly associated with ALS, its incomplete penetrance—reflecting considerable interindividual variability—indicates that additional proteins and pathways likely modify the progression from carrier status to symptomatic disease [10].

The comprehensive plasma proteomic profiling resource from the UK Biobank offers a unique opportunity to meticulously investigate proteomic alterations during the decade preceding ALS onset. Therefore, in this prospective analysis of 52 584 participants, we assessed the association between baseline plasma levels of 2920 proteins and ALS outcomes using multivariate Cox proportional hazards (PH) models. Subgroup analysis was subsequently stratified by *C9orf72* HRE status. Then, the disease prediction model was employed to assess the performance of candidate proteins in predicting the onset of ALS. Further analyses examined the relationship between levels of identified proteins and both age at onset (AAO) and survival in ALS patients. Finally, we investigated cross‐sectional temporal patterns of plasma proteins in ALS, performed Mendelian randomization (MR) and colocalization analysis to evaluate potential causal relationships between identified proteins and disease and conducted functional enrichment analysis to explore potential pathogenic mechanisms of ALS. A schematic overview of the study design is presented in Figure 1.

**FIGURE 1:**
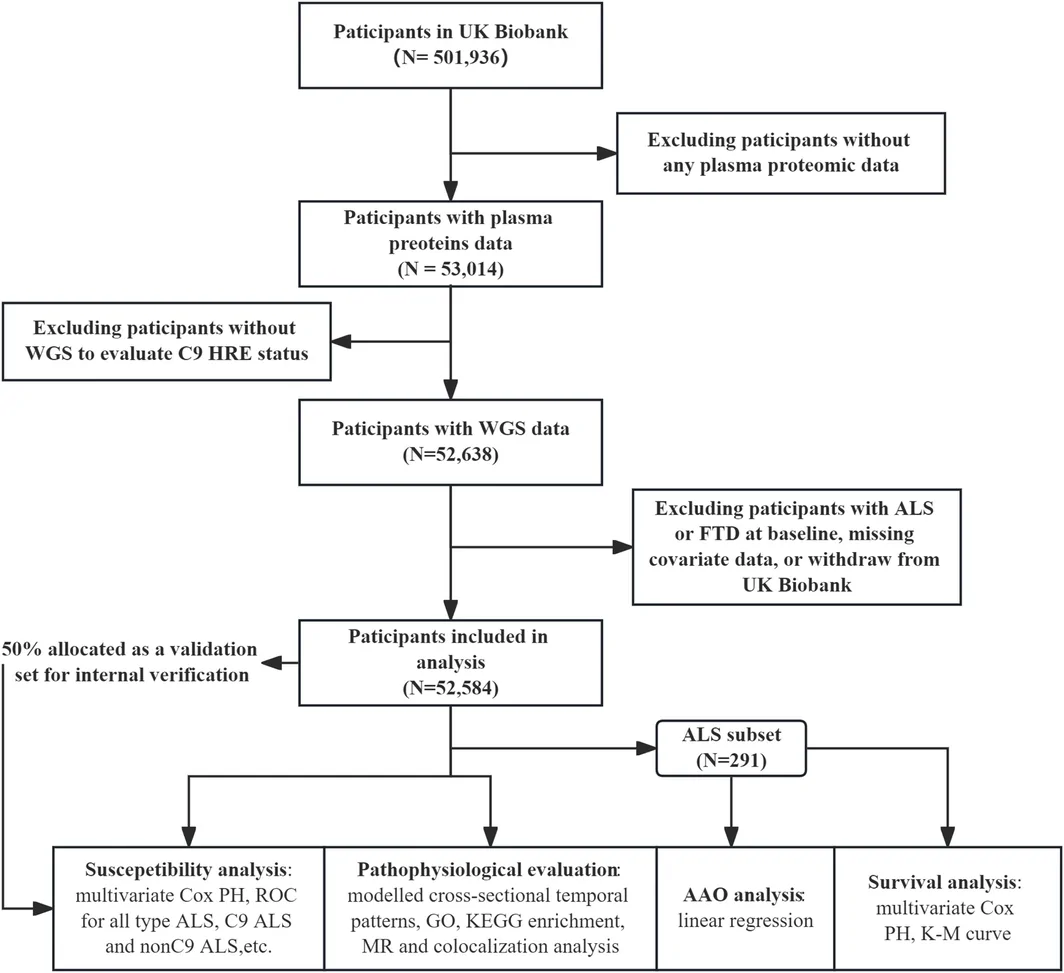
Flowchart of the study design and workflow. Abbreviations: AAO, age at onset; ALS, amyotrophic lateral sclerosis; C9 HRE, *C9orf72* hexanucleotide repeat expansion; Cox PH, Cox proportional hazards models; FTD, frontotemporal dementia; GO, Gene Ontology; KEGG, Kyoto Encyclopedia of Genes and Genomes; K–M, Kaplan–Meier; MR, Mendelian randomization; ROC, receiver operating characteristic; WGS, whole‐genome sequencing.

## 2 Methods

### 2.1 Study Population

The UK Biobank recruited more than 500 000 people aged 40–69 from the general public during the 2006–2010 enrollment period [11]. From an initial cohort of 53 014 participants with available proteomic data, we excluded those with a baseline diagnosis of ALS or frontotemporal dementia (FTD), missing covariate information, study withdrawal or lack of whole‐genome sequencing (WGS) data. The final analytical sample comprised 52 584 participants.

### 2.2 Blood Proteomics

Using the Olink platform, a total of 2923 proteins were assayed in the UK Biobank. Of these, three proteins—GLIPR1 (glioma pathogenesis‐related protein 1), NPM1 (nucleophosmin) and PCOLCE (procollagen C‐endopeptidase enhancer 1)—were discarded from the analytical dataset owing to a missing data rate exceeding 50%, ensure overall data quality for subsequent analyses. Consequently, a total of 2920 unique proteins (Table S1) meeting the predefined quality control criteria were included in the final dataset. However, as not all participants had complete proteomic data, the analysis for each protein was restricted to the subset of participants with available measurements for that specific protein. More detail is shown in the Supporting Information.

### 2.3 Estimation of C9orf72 HRE Status

Employing Illumina NovaSeq technology, the UK Biobank performed WGS on blood samples obtained from more than 490 000 participants. In line with our previous work [12], *C9orf72* HRE carriers were identified using *ExpansionHunter* [13], a validated algorithm tailored for the detection of pathogenic repeat expansions in short tandem repeat (STR) regions, including the *C9orf72* HRE. The performance of this method in detecting *C9orf72* HRE was benchmarked against the gold standard repeat‐primed PCR (RP‐PCR), yielding 100% sensitivity and 99.8%–99.9% specificity [13]. Based on clinical and genetic evidence from prior studies [14], a pathogenicity threshold for *C9orf72* HRE was defined as > 30 repeat expansions. Individuals meeting this threshold were classified as carriers of *C9orf72* HRE.

### 2.4 Determination of ALS Incidence, AAO and Survival

The primary endpoint was the occurrence of ALS. Incident ALS cases were delineated as those diagnosed after the baseline visit, date (coincident with blood sample collection). Follow‐up began on the date of each participant's initial assessment centre visit and extended until the first occurrence of any of the following: a recorded diagnosis of ALS, death, loss to follow‐up or the study censoring date (September 2023). The AAO of ALS was defined as the time from the participant's date of birth to the date of the first reported ALS diagnosis. Survival duration was calculated as the period from the first reported ALS diagnosis to the date of death.

### 2.5 Statistical Analysis

The Cox PH models were employed to identify proteins associated with incident ALS, adjusting for potential confounders (Supporting Information). Significant associations were determined using FDR‐adjusted (false discovery rate) *p*‐values (Benjamini–Hochberg method), with a threshold of < 0.05. Hazard ratios (HRs) and corresponding 95% confidence intervals (CIs) were calculated to assess the magnitude and direction of the associations. Subgroup analysis was performed to evaluate proteins linked to *C9orf72*‐related ALS (C9‐ALS) and non‐*C9orf72* ALS (non‐C9‐ALS) separately within the general ALS population. To pinpoint C9orf72‐specific associations, the Cox PH models were further applied exclusively to the subgroup of *C9orf72* HRE carriers. Linear regression analysis was conducted to explore associations between plasma protein levels and AAO of ALS, with subsequent subgroup analyses conducted for C9‐ALS and non‐C9‐ALS cases. The associations between plasma protein expression and ALS survival were assessed using the Cox PH models and the Kaplan–Meier analysis. Proteomic comparisons between prediagnostic and symptomatic ALS were performed using ANCOVA (analysis of covariance), adjusting for multiple covariates, with significance at FDR < 0.05 (more detail in the Supporting Information). To predict ALS occurrence at 3, 5 and 10 years, the identified proteins were integrated with age and gender. Time‐dependent receiver operating characteristic (ROC) curves were constructed for each time point. Model performance was assessed via time‐dependent area under the curve (AUC) with 95% CI, Harrell's concordance index (C‐index), integrated Brier score, optimism‐adjusted calibration slope and expected‐to‐observed (E/O) event ratio, calculated separately in the full cohort and the internal validation cohort (full methods in the Supporting Information). As an exploratory analysis, we expanded the signature with additional proteins meeting *p* < 0.01 in the Cox PH analysis, with all related findings interpreted as hypothesis‐generating.

Moreover, detailed methodologies for covariates, assessment of cross‐sectional temporal patterns for ALS‐associated proteins, enrichment analysis, MR, colocalization and sensitivity analysis are also provided in the Supporting Information.

## 3 Results

### 3.1 Population Characteristics

The analysis comprised 52 584 participants without ALS at baseline and with available proteomics data. The cohort had a mean age of 56.8 years (SD: 8.2), was 53.9% female and 93.3% White. During a mean follow‐up period of 11.8 years, 291 incident ALS cases were documented, 34 of which carried a *C9orf72* HRE of > 30 repeats. Half of the study population (*n* = 26 292) was randomly allocated to an internal validation cohort, in which 136 incident ALS cases were observed during follow‐up. Table 1 shows baseline characteristics by ALS status; detailed characteristics for the validation sets are provided in Table S2.

**TABLE 1:** Baseline characteristics of the study participants from the UK Biobank cohort.

Variants	Features	Overall (*n* = 52 584)	Non‐ALS (*n* = 52 293)	All incident ALS (*n* = 291)	*p*	Incident non‐C9‐ALS (*n* = 257)	Incident C9‐ALS (*n* = 34)
Age, years^a^		56.807 ± 8.211	56.787 ± 8.214	60.464 ± 6.837	< 0.001	60.825 ± 6.875	57.735 ± 5.946
Sex^b^	Male	24 218 (46.1%)	24 060 (46.0%)	158 (54.3%)	0.006	147 (57.2%)	11 (32.4%)
	Female	28 366 (53.9%)	28 233 (54.0%)	133 (45.7%)		110 (42.8%)	23 (67.6%)
Ethnicity^b^	White	49 041 (93.3%)	48 761 (93.2%)	280 (96.2%)	0.057	249 (96.9%)	31 (91.2%)
	Others	3543 (0.7%)	3532 (6.8%)	11 (3.8%)		8 (3.1%)	3 (8.8%)
TDI^a^		−1.151 ± 3.185	−1.181 ± 3.185	−1.235 ± 3.123	0.773	−1.217 ± 3.167	−1.371 ± 2.808
BMI (kg/m^2^)^a^		27.469 ± 4.786	27.787 ± 4.787	27.580 ± 4.533	0.693	27.741 ± 4.513	26.358 ± 4.563
Smoking status^b^	Current	5553 (10.6%)	5523 (10.6%)	30 (10.3%)	0.343	26 (10.1%)	4 (11.8%)
	Previous	18 329 (34.8%)	18 213 (34.8%)	116 (39.9%)		106 (41.2%)	20 (58.8%)
	Never	28 511 (54.2%)	28 367 (54.2%)	144 (49.5%)		124 (48.2%)	10 (29.4%)
	Prefer not to answer	191 (0.4%)	190 (0.4%)	1 (0.3%)		1 (0.4%)	0
Alcohol intake frequency^b^	Daily or almost daily	10 602 (20.2%)	10 529 (20.1%)	73 (25.1%)	0.271	66 (25.7%)	7 (20.6%)
	Three or four times a week	11 818 (22.5%)	11 766 (22.5%)	52 (17.9%)		45 (17.5%)	7 (20.6%)
	Once or twice a week	13 646 (26.0%)	13 573 (26.0)	73 (25.1%)		64 (24.9%)	9 (26.5%)
	One to three times a month	5745 (10.9%)	5715 (10.9%)	39 (13.4%)		25 (9.7%)	5 (14.7%)
	Special occasions only	6166 (11.7%)	6127 (11.7%)	39 (13.4%)		36 (14.0%)	3 (8.8%)
	Never	4544 (8.6%)	4520 (8.6%)	24 (8.2%)		21 (8.2%)	3 (8.8%)
	Prefer not to answer	63 (0.1%)	63 (0.1%)	0		0	0
Sample age, years^a^		12.718 ± 0.851	12.717 ± 0.850	12.730 ± 0.884	0.801	12.769 ± 0.883	12.436 ± 0.846
Fasting time, hours^a^		3.838 ± 2.493	3.838 ± 2.495	3.732 ± 2.121	0.469	3.739 ± 2.082	3.676 ± 2.434
*C9orf72* HRE status^b^	> 30 repeats	128 (0.2%)	94 (0.2%)	34 (11.7%)	< 0.001	0	34 (100%)
	≤ 30 repeats	52 456 (99.8%)	52 199 (99.8%)	257 (88.3%)		257 (100%)	0
Follow‐up time, years^a^		11.812 ± 3.448	11.856 ± 3.412	5.845 ± 3.063		5.969 ± 3.041	4.945 ± 3.125

*Note:* Data are presented as *n* (%) for categorical variables and mean ± standard deviation (SD) for continuous variables, as appropriate. Group comparisons were performed using Student's *t*‐test (for normally distributed continuous variables) or the Mann–Whitney *U* test (for nonnormally distributed continuous variables) and the *χ* ^2^‐test for categorical variables. All tests were two‐sided.

^a^ Mean ± SD.

^b^ *n* (%).

### 3.2 Association Between Protein and All‐Type Incident ALS Risk

We applied the Cox PH models to identify plasma proteins associated with incident all‐type ALS, with adjustments for age, sex, ethnicity, townsend deprivation index (TDI), body mass index (BMI), smoking status, alcohol intake frequency, fasting duration and sample age as potential confounders based on previous studies. Of the 2920 proteins analysed, nine demonstrated significant associations with ALS risk following FDR adjustment (Figure 2a,b and Table S3). Plasma NEFL (neurofilament light polypeptide) demonstrated the strongest association with ALS risk (HR: 2.897, 95% CI: 2.566–3.271, FDR < 0.001), followed by EGFLAM (Pikachurin; HR: 0.402, 95% CI: 0.266–0.606, FDR = 0.020), CSRP3 (cysteine and glycine rich protein 3) (HR: 1.233, 95% CI: 1.116–1.362, FDR = 0.022), MYBPC1 (Myosin binding protein C slow type) (HR: 1.352, 95% CI: 1.171–1.561, FDR = 0.022), MEGF10 (Multiple epidermal growth factor like domains protein 10) (HR: 1.744, 95% CI: 1.336–2.276, FDR = 0.022), ITGAV (Integrin alpha V) (HR: 0.284, 95% CI: 0.155–0.520, FDR = 0.022), MYOM3 (Myomesin 3) (HR: 1.289, 95% CI: 1.140–1.458, FDR = 0.022), THBS4 (Thrombospondin 4) (HR: 1.570, 95% CI: 1.251–1.969, FDR = 0.035) and CD248 (Endosialin) (HR: 0.573, 95% CI: 0.430–0.763, FDR: 0.044). We further assessed the predictive performance of a model integrating these nine identified proteins with age and sex for ALS onset at 3, 5 and 10 years. This integrated model produced AUC values exceeding 0.78 across all time points, with the highest performance observed at the 3‐year interval (AUC: 0.867, 95% CI: 0.826–0.924; Figure 2c). In the internal validation set, NEFL, CD248, EGFLAM, ITGAV and MYBPC1 remained significantly associated with ALS risk (Table S4). Baseline NPX levels of the 9 ALS‐associated proteins in patients with ALS and controls are presented in Figure S1. The model demonstrated superior performance compared to the model only using age and sex (Figure S2). Furthermore, the model demonstrated good performance in the internal validation cohort (Figure S3), and an exploratory model for predicting all‐type incident ALS risk is presented in Figure S4.

**FIGURE 2:**
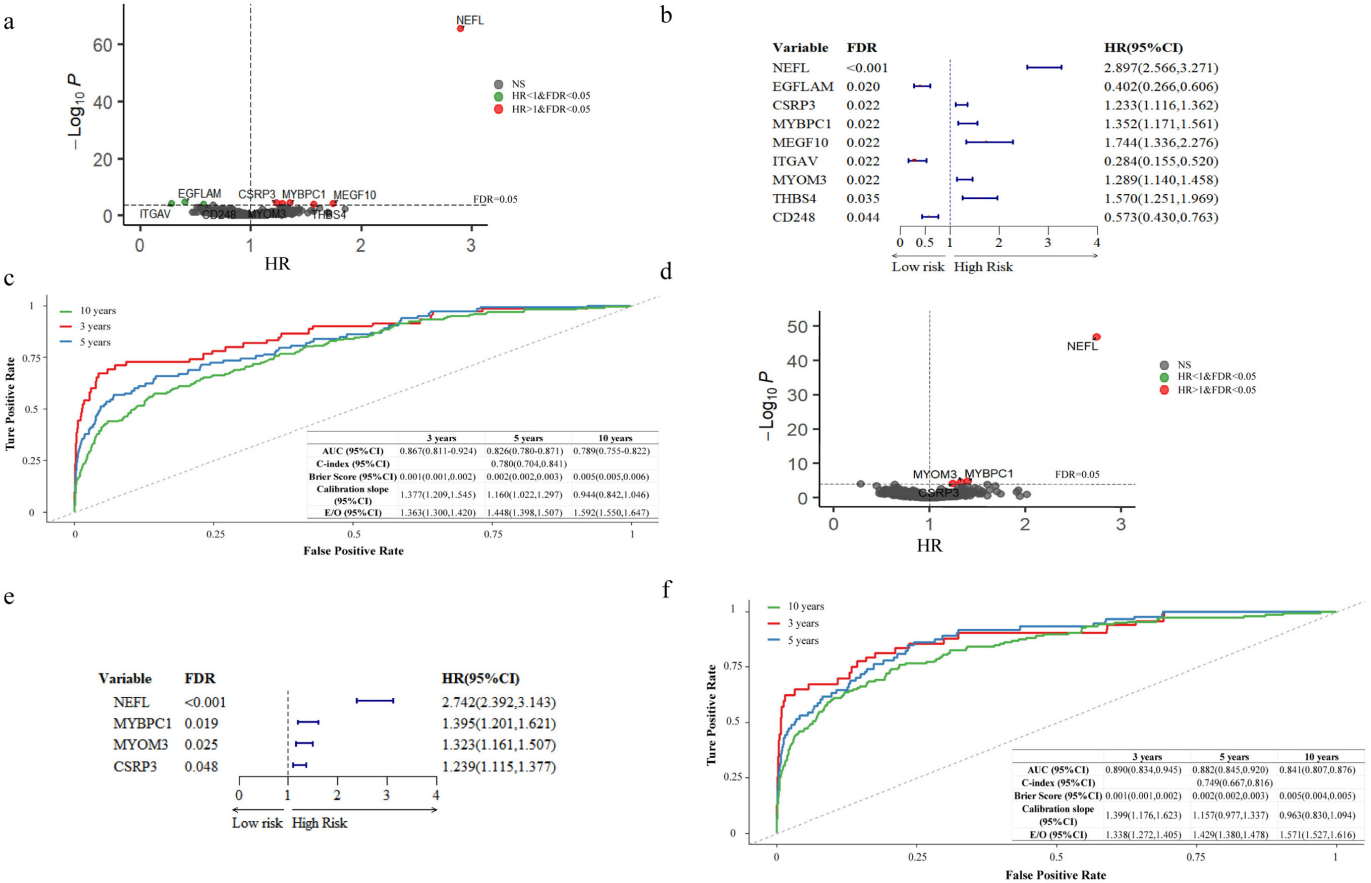
Plasma proteins associated with incident ALS and their predictive performance. (a) Volcano plots displaying the HR (*x*‐axis) and statistical significance (−log10 of two‐sided *p*‐values, *y*‐axis) for the associations of standardized protein abundance with incident all‐type ALS. (b) Forest plot of significantly all‐type ALS‐associated proteins (FDR‐corrected). (c) ROC using identified proteins, age and sex to predict all‐type ALS incidence at 3, 5 and 10 years in discovery cohort. (d) Volcano plots displaying the HR (*x*‐axis) and statistical significance (−log10 of two‐sided *p*‐values, *y*‐axis) for the associations of standardized protein abundance with incident non‐C9‐ALS. (e) Forest plot of significantly non‐C9‐ALS‐associated proteins (FDR‐corrected). (f) ROC using identified proteins, age and sex to predict non‐C9‐ALS incidence at 3, 5 and 10 years in discovery cohort.

### 3.3 Association Between Protein and Incident Non‐C9‐ALS Risk

To assess the specific association of plasma proteins with non‐C9‐ALS, we first excluded cases carrying the *C9orf72* mutation. Four proteins were significantly associated with non‐C9‐ALS risk (Figure 2d,e and Table S5). NEFL emerged as the strongest predictor of non‐C9‐ALS risk (HR: 2.742, 95% CI: 2.392–3.143). Subsequent significant associations were observed for MYBPC1 (HR: 1.395, 95% CI: 1.201–1.621), MYOM3 (HR: 1.323, 95% CI: 1.161–1.507) and CSRP3 (HR: 1.239, 95% CI: 1.115–1.377). The associations of NEFL and MYBPC1 with non‐C9‐ALS risk were further validated in the internal validation set (Table S6). We assessed the predictive performance of a model incorporating age and sex along with the four identified proteins for non‐C9‐ALS onset at 3, 5 and 10 years. An AUC of 0.808 was achieved by the integrated model for the 3‐year prediction, while all analyses yielded AUCs greater than 0.78 (Figure 2f). Figure S5 shows the baseline NPX levels of the four non‐C9‐ALS associated proteins in ALS patients and controls. Our final model significantly outperformed the age‐and‐sex‐only baseline model (Figure S6). Figures S7 and S8 depict the predictive performance in the internal validation cohort and the exploratory model for incident non‐C9‐ALS risk, respectively.

### 3.4 Association Between Protein and Incident C9‐ALS Risk

To identify plasma proteins specifically associated with the risk of C9‐ALS, we performed the Cox PH models comparing C9‐ALS patients with healthy controls. A strong association was observed between NEFL and C9‐ALS (HR = 4.043, 95% CI: 3.069–5.327). Additionally, COL5A1 was also significantly associated with C9‐ALS incidence (HR = 3.072, 95% CI: 2.124–4.444) (Figure S9a,b and Table S7). Both of them were validated in the internal validation set (Table S8). For predicting C9‐ALS onset, the highest performance was achieved by the integrated model (NEFL, COL5A1, age and sex) at the 3‐year time point (AUC = 0.955, 95% CI: 0.902–1.000), with AUCs exceeding 0.77 across all evaluated time points (Figure S9c). Figure S10 shows the baseline NPX levels of the two C9‐ALS‐associated proteins in ALS patients and controls. When compared to a model based solely on age and sex, our integrated model exhibited superior predictive accuracy (Figure S11). The internal validation profiles and the exploratory incident C9‐ALS prediction model are displayed in Figures S12 and S13.

### 3.5 Association Between Protein and Incident C9‐ALS Risk in C9orf72 HRE Carriers

We further analysed a subcohort of *C9orf72* HRE carriers, given that not all carriers develop ALS. Our cohort included 128 participants carrying *C9orf72* HREs (expansion size > 30 repeats), and their baseline characteristics stratified ALS status are shown in Table S9. While ALS‐affected individuals showed a larger median expansion size than non‐ALS controls (*p* = 0.012), this association was not absolute: carriers with 30–100 repeats developed ALS, whereas some individuals with thousands of repeats remained free of the disease (Figure 3a). No statistically significant association was observed between *C9orf72* HRE size and age at ALS onset (Figure 3b). Furthermore, among *C9orf72* HRE carriers, the Cox PH model was applied to identify potential modifiers of ALS risk or biomarkers indicative of the transition from a non‐ALS to an ALS state. Among all tested proteins, only NEFL remained significantly associated with ALS risk following FDR correction, with elevated baseline NEFL levels predicting subsequent ALS onset (HR = 3.378, 95% CI: 1.954–5.839, Figure 3c and Table S10). Carriers who subsequently developed ALS exhibited significantly higher baseline NEFL levels compared to nonprogressors (Figure 3d). However, no significant linear correlation was observed between *C9orf72* repeat expansion size and plasma NEFL levels (adjusted *p* = 0.080, Figure S14).

**FIGURE 3:**
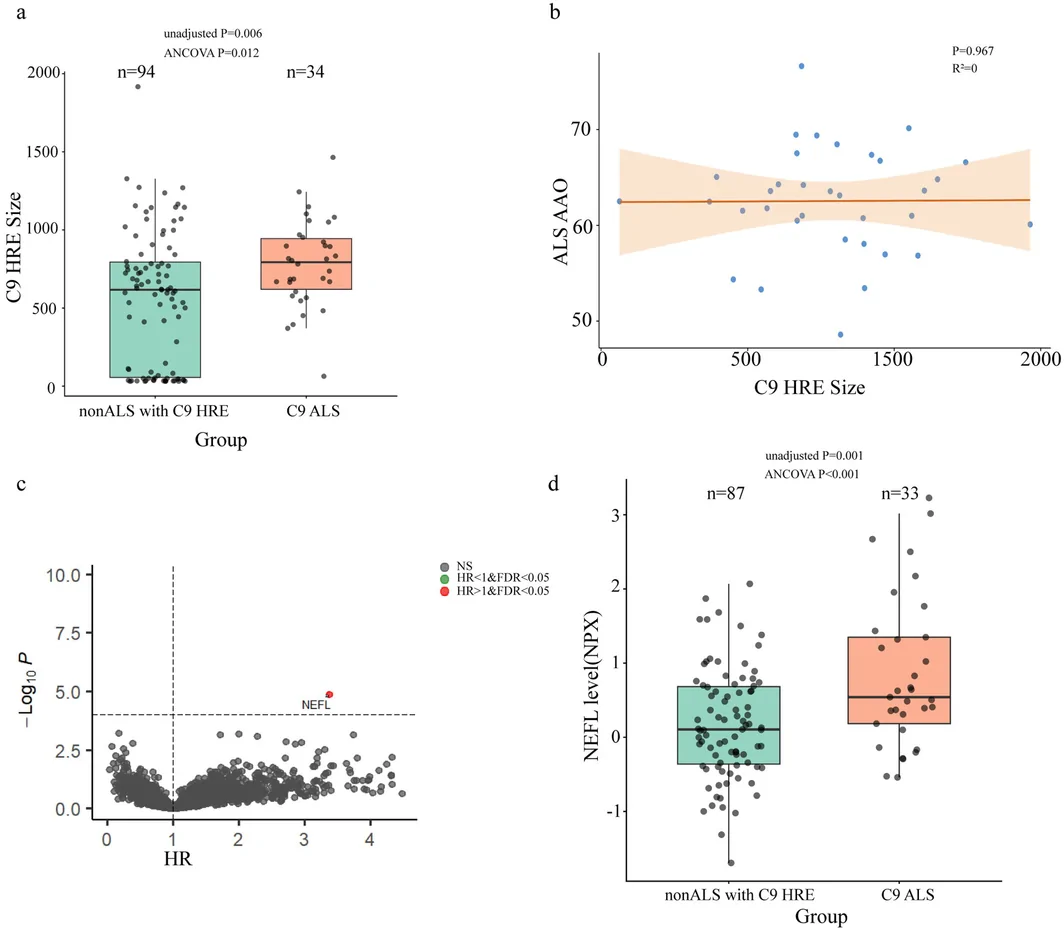
ALS‐related protein in *C9orf72* HRE carriers. (a) Distribution of amplification times in *C9orf72* HRE carriers, comparing ALS patients and non‐ALS controls. Box‐and‐scatter plot showing median (central horizontal line), interquartile range (IQR; box boundaries at 25th and 75th percentiles) and individual data points. Statistical analysis was performed using the Wilcoxon rank‐sum test (for nonnormally distributed data) with adjustment for sex and age as covariates via analysis of covariance (ANCOVA); *p* < 0.05 was considered statistically significant. (b) Relationship between the amplification times of *C9orf72* HRE and AAO in C9‐ALS patients. A linear regression model was fitted to quantify the association (solid line: fitted regression line; shaded area: 95% confidence interval for the regression slope). Statistical inferences were derived directly from the linear regression framework. *p* < 0.05 was considered statistically significant. (c.) Volcano plots displaying the HR (*x*‐axis) and statistical significance (−log10 of two‐sided *p*‐values, *y*‐axis) for the associations of standardized protein abundance with incident C9‐ALS in *C9orf72* HRE carriers. (d) Distribution of plasma NEFL level in *C9orf72* HRE carriers, comparing ALS patients and non‐ALS controls. Box‐and‐scatter plot showing median (central horizontal line), interquartile range (IQR; box boundaries at 25th and 75th percentiles) and individual data points. Statistical analysis was performed using the Wilcoxon rank‐sum test (for nonnormally distributed data) with adjustment for sex and age as covariates via analysis of covariance (ANCOVA); *p* < 0.05 was considered statistically significant.

### 3.6 Association Between Protein and ALS Onset Age

The mean AAO was 65.967 years (SD: 7.797) for all‐type ALS, 66.422 years (SD: 7.940) for non‐C9‐ALS and 62.534 years (SD: 5.608) for C9‐ALS patients. Although unadjusted analysis indicated an earlier AAO in C9‐ALS compared to non‐C9‐ALS, this difference was no longer statistically significant after adjustment for multiple confounding factors (Figure S15). We examined the association between prediagnostic plasma protein levels and AAO of ALS using linear regression after adjusting age, sex, ethnicity, TDI, BMI, smoking status, frequency of alcohol intake, fasting time and sample age. Higher plasma NEFL levels were associated with a younger AAO in all‐type ALS (*β* = −1.501, FDR < 0.001) and non‐C9‐ALS (*β* = −1.440, FDR < 0.001) (Figure 4a–d, Tables S11 and S12), whereas no significant association was observed for C9‐ALS (*β* = −1.159, FDR = 0.467) (Table S13). SSC5D (soluble scavenger receptor cysteine rich domain containing protein) showed a potential association with AAO (*β* = 2.691), but it is not statistically significant (FDR = 0.178) (Figure 4e,f and Table S13).

**FIGURE 4:**
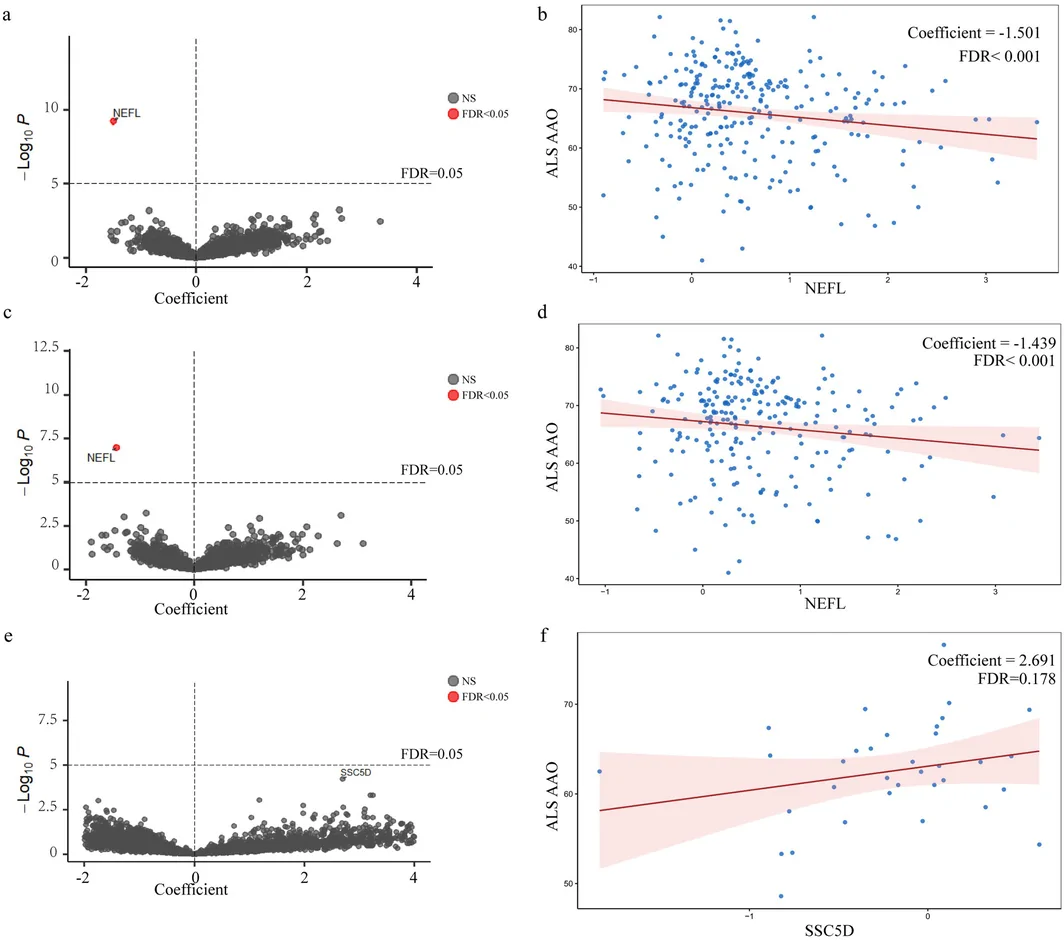
Plasma proteins associated with ALS onset age. (a) Volcano plots displaying the coefficient (*x*‐axis) and statistical significance (−log10 of two‐sided *p*‐values, *y*‐axis) for the associations of standardized protein abundance with incident all‐type ALS. (b) Linear regression plot showing relationship between NFEL and all‐type ALS AAO. (c) Volcano plots displaying the coefficient (*x*‐axis) and statistical significance (−log10 of two‐sided *p*‐values, *y*‐axis) for the associations of standardized protein abundance with incident non‐C9ALS. (d) Linear regression plot showing relationship between NFEL and non‐C9ALS AAO. (e) Volcano plots displaying the coefficient (*x*‐axis) and statistical significance (−log10 of two‐sided *p*‐values, *y*‐axis) for the associations of standardized protein abundance with incident C9‐ALS. (f) Linear regression plot showing relationship between SSC5D and C9‐ALS AAO.

### 3.7 Association Between Protein and ALS Survival

To evaluate the association between pre‐diagnostic plasma protein levels and ALS survival, we employed the Cox proportional hazards models adjusted for the aforementioned covariates. The mean survival time was 2.518 years (SD: 2.852) for all‐type ALS patients, compared with 2.637 years (SD: 2.968) in non‐C9‐ALS and 1.624 years (SD: 1.479) in C9‐ALS patients. The Kaplan–Meier analysis demonstrated a significantly shorter overall survival for patients with C9‐ALS compared to those with non‐C9‐ALS (log‐rank test, *p* = 0.048, Figure S16). After FDR correction, no significant associations between prediagnostic plasma proteins and survival were observed in either the all‐type or non‐C9‐ALS cohorts (Tables S14 and S15). We identified 33 candidate proteins potentially associated with survival in C9‐ALS patients (FDR < 0.05; Table S16). However, the limited sample size necessitates cautious interpretation, and the global Schoenfeld test indicated potential violations of the PH assumption across multiple covariates (Table S16).

### 3.8 Modelled Cross‐Sectional Temporal Patterns of ALS‐Associated Proteins

A total of 14 proteins were classified as abnormal (defined by an average absolute *Z*‐score ≥ 0.20) during the prediagnostic period (Figure 5a and Table S17). Subsequent analysis of their expression levels across sectional temporal intervals revealed that numerous ALS‐associated proteins—such as NEFL, MYOM3, MEGF10, CSRP3, MYBPC1, THBS4, ITGAV, CD248 and EGFLAM—displayed significant alterations (either elevated or reduced). In contrast, other proteins, including CAPN3, MCEE, HS6ST2, SHBG and VSIG10, while not reaching statistical significance in time‐to‐event analysis, still exhibited dysregulated expression prior to clinical onset. To comprehensively analyse the cross‐sectional temporal patterns of ALS‐associated proteins, we expanded our cohort to incorporate participants diagnosed with ALS before baseline (*n* = 45). The clinical characteristics of this expanded subgroup are detailed in Table S18. Figure 5b depicts the cross‐sectional temporal patterns of NEFL levels in relation to the time of diagnosis (prediagnosis and postdiagnosis) for all ALS patients, fitted using a GAM. Plasma NEFL levels in ALS patients begin a gradual increase approximately 9.27 years before diagnosis, reaching a peak 1–2 years prior to diagnosis specifically in the non‐C9‐ALS subgroup. In contrast to non‐C9‐ALS patients, no distinct peak in plasma NEFL levels was observed in the C9‐ALS cohort; rather, these individuals exhibited a more pronounced rate of NEFL increase compared to non‐C9‐ALS cases. Temporal expression patterns of additional ALS‐related proteins are shown in Figure S17. Furthermore, cross‐sectional ANCOVA identified five differentially expressed proteins (ALDH3A1, GALNT5, RBFOX3, CHCHD6 and CORO6; Figure S18) between prediagnostic ALS patients and symptomatic ALS before baseline. However, none of the candidate risk proteins from the Cox PH achieved significance in this comparison (Figure S19). Table S19 presents cross‐sectional ANCOVA results comparing protein levels in prediagnostic versus symptomatic ALS stages.

**FIGURE 5:**
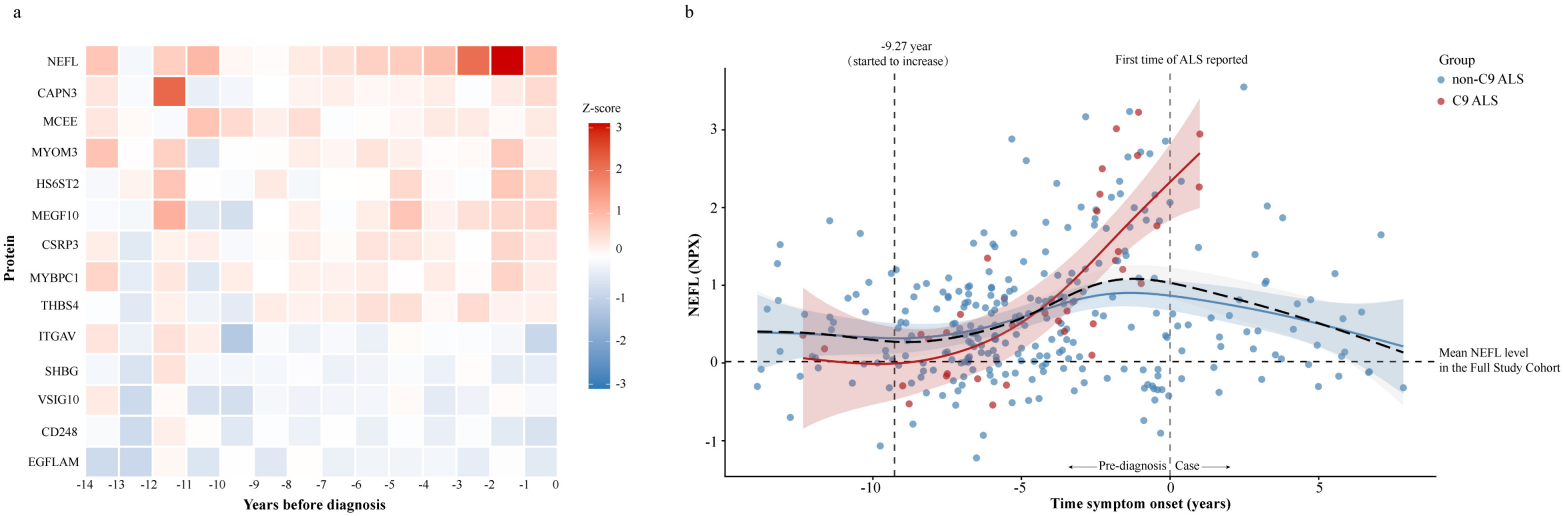
Temporal evolutions of plasma proteins across the ALS course (prediagnosis and postdiagnosis). (a) The heatmap of proteins with significant abundance changes (mean absolute *Z*‐score threshold > 0.2). Using matched control samples as a reference (mean and SD; see Section 2), protein levels were *Z*‐scored. For each protein and year, the displayed *z*‐score is the average of all individual measurements from that period. (b.) Plasma NEFL cross‐sectional temporal patterns in ALS patients. Each dot represents one patient (red: C9‐ALS, blue: non‐C9‐ALS). Fitted curves were generated using generalized additive models (GAM). The solid lines indicate model fits for C9‐ALS (red) and non‐C9‐ALS (blue) cohorts, while the black dotted line represents the fit for the entire ALS cohort. The shaded area denotes the 95% confidence interval for the overall fit.

### 3.9 Biological Function of ALS‐Associated Proteins

To elucidate the biological functions and regulatory pathways associated with the identified ALS‐linked proteins (nominal *p* < 0.05), a functional enrichment analysis was conducted (Tables S20–S22). The analysis revealed an enrichment of several muscle‐related pathways, including actomyosin structure organization and muscle cell development (Figure 6a). The enrichment profile for non‐C9‐ALS closely mirrored that of all‐type ALS (Figure 6b–d). For the C9‐ALS associated proteins, the enrichment analysis yielded slightly different results (Figure 6e,f), and despite this difference, several KEGG pathways were common across all three groups, notably the cell adhesion molecules and PPAR signalling pathways. Furthermore, we investigated the potential pathways associated with the transition from a non‐ALS to an ALS state in *C9orf72* HRE carriers and identified immune receptor‐ and cytokine receptor‐related interactions as the most prominent [15] (Figure S20 and Table S23).

**FIGURE 6:**
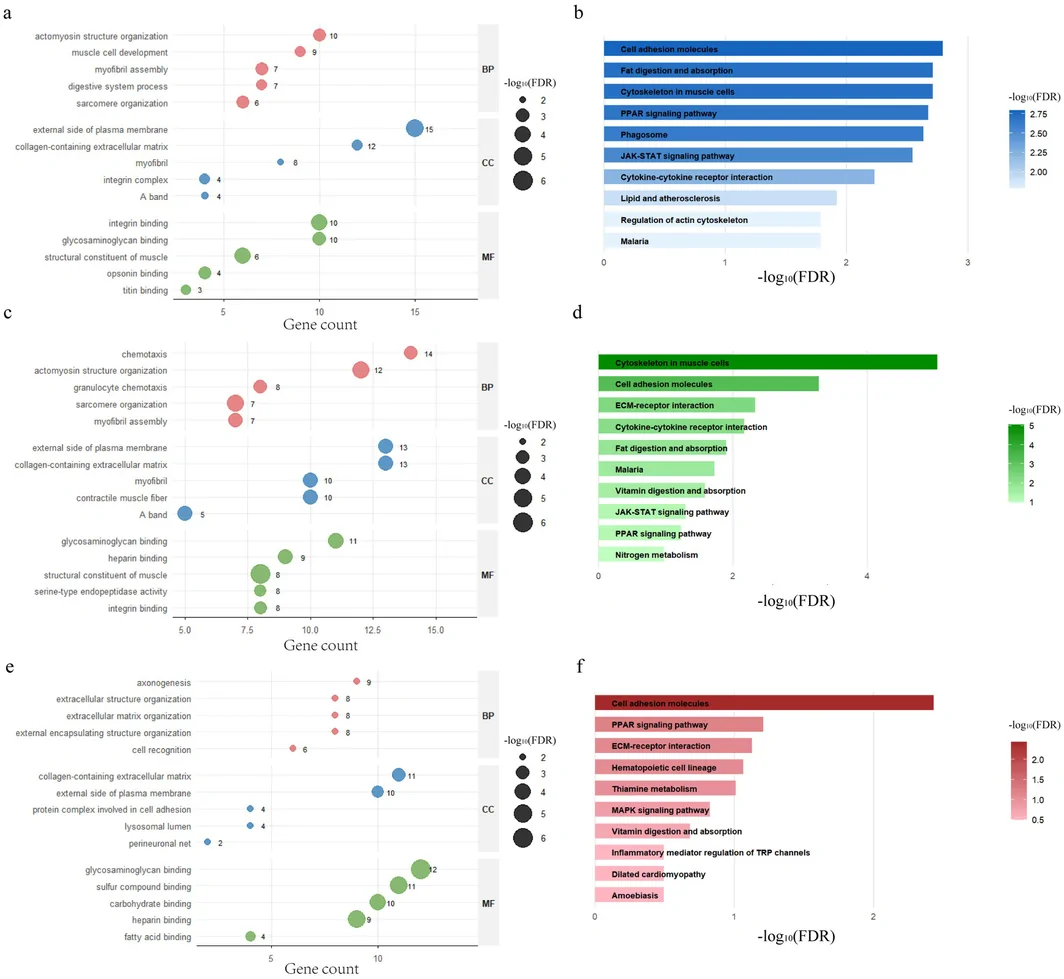
GO and KEGG enrichment analysis of proteins nominally associated with ALS. (a) Top 5 enriched GO terms for all‐type ALS‐associated proteins (nominal *p* < 0.05). Results are displayed separately for the three GO categories: Biological process (BP), cellular component (CC) and molecular function (MF). (b) Top 10 enriched KEGG pathways for all‐type ALS‐associated proteins (nominal *p* < 0.05). (c) Top 5 enriched GO terms for non‐C9‐ALS‐associated proteins (nominal *p* < 0.05). Results are displayed separately for the three GO categories: BP, CC and MF. (d) Top 10 enriched KEGG pathways for non‐C9‐ALS‐associated proteins (nominal *p* < 0.05). (e) Top five enriched GO terms for C9‐ALS‐associated proteins (nominal *p* < 0.05). Results are displayed separately for the three GO categories: BP, CC and MF. f. Top 10 enriched KEGG pathways for C9‐ALS‐associated proteins (nominal *p* < 0.05).

### 3.10 Assessing Causal Plasma Protein Targets in ALS via MR and Colocalization Analysis

Using pQTLs as instrumental variables, we performed MR to estimate putative causal effects on ALS risk for the 10 proteins previously identified as significant in the Cox PH analysis. A total of 202 SNPs were selected as valid IVs for these proteins (Table S24). The MR analysis indicated that elevated genetically predicted levels of MYOM3 were nominally associated with an increased risk of ALS (odds ratio: 1.101, 95% CI: 1.003–1.210, *p* = 0.043; Table S25). Sensitivity analysis was further conducted to evaluate potential heterogeneity and pleiotropy (Tables S26 and S27). Colocalization analysis revealed no significant evidence for shared causal variants between the protein pQTLs and ALS GWAS signals (all PPH4 < 0.8) (Table S28), and the colocalization plot revealed a lack of shared causal variants (Figure S21).

### 3.11 Sensitivity Analysis

The primary association remained largely consistent across all age and sex subgroups (Table S29). EGFLAM, ITGAV and THBS4 exhibited a stronger association with ALS in the subgroup of patients younger than 60 years old (Table S29). Sensitivity analysis employing different lag periods (1‐, 3‐ and 5‐year intervals) produced relatively consistent results (Table S30). NEFL and MEGF10, which met FDR < 0.05 significance in the primary ALS risk model and exhibited only mild time‐varying effects (global PH test *p* < 0.001; mild log‐hazard attenuation on Schoenfeld residuals; Figures S22 and S23), are summarized by their conventional full‐follow‐up HRs, supplemented by period‐specific HRs at 0–3, 0–5, and 0–10 years (Figures S22 and S23). The direction and magnitude of these period‐specific HRs were broadly consistent with the conventional full‐follow‐up estimate, indicating that mild time‐varying effects do not alter the overall interpretation of the primary association.

## 4 Discussion

To date, the long‐standing pursuit of a reliable method for identifying presymptomatic biomarkers and characterizing prodromal stages of ALS has begun to yield unprecedented opportunities for earlier diagnosis, therapeutic intervention and potential prevention in both familial and sporadic forms of the disease. However, the search for biomarkers in ALS has historically focused on the analysis of individual biomarkers, such as NEFL [16]. In a cross‐sectional analysis, Chia et al. [3] identified a plasma proteomic biomarker panel for ALS (*n* = 183) and observed its correlation with AAO in a subset of individuals (*n* = 110). Nevertheless, the cross‐sectional design of this study necessitates longitudinal validation to establish the predictive utility of these biomarkers. Our prospective cohort data substantiate the link between NEFL and ALS, showing that its predictive power as a biomarker is not merely confined to the reported 12‐month window [17] and significantly precedes clinical manifestation by as much as a decade, irrespective of *C9orf72* HRE status. Notably, in alignment with our observation of a significantly accelerated NEFL elevation rate in C9‐ALS relative to non‐C9‐ALS, a recent study also suggests that C9‐ALS is characterized by higher blood NEFL levels [18]. Previous investigation had established that NEFL concentrations exhibit a progressive increase prior to clinical diagnosis, followed by stabilization post‐diagnosis [19]. In our analysis, while non‐C9‐ALS patients exhibited a distinct pattern with NEFL peaking 1–2 years before diagnosis, this observation may be attributable to the absence of repeated longitudinal measurements within the same individuals. In contrast, C9‐ALS patients exhibited divergent cross‐sectional temporal patterns—consistent with prior work [20]—marked by a gradual elevation in NEFL without a prediagnostic peak. Furthermore, after adjusting for potential confounders, our analysis confirmed that higher pre‐onset plasma NEFL levels independently predict an earlier AAO in non‐C9‐ALS, consistent with previous report [3]. However, the modest sample size of the C9‐ALS cohort limited the ability to detect significant associations between NEFL (or other proteins) and the AAO in C9‐ALS. Despite the well‐documented association of NEFL with ALS survival [21], our analysis indicated no significant correlation between prediagnostic plasma NEFL levels and patient prognosis. In C9‐ALS subgroup, several prediagnostic protein levels showed associations with survival, while these observations should be considered preliminary and interpreted with caution owing to the limited sample size of C9‐ALS.

Beyond the identification of NEFL, our findings corroborate several additional proteins previously highlighted in a cross‐sectional investigation [3], including CSRP3, MYBPC1, MYOM3 and MEGF10. CSRP3, also referred to as muscle LIM protein (MLP), plays a fundamental role in muscle development and in maintaining the structural integrity of muscle cells [22] and facilitates proper autophagosome formation via its interaction with LC3 [23]. Nevertheless, whether CSRP3 participates in mediating autophagy dysregulation in ALS remains an open question. Additionally, MYBPC1 and MYOM3 are also muscle‐related proteins. A definitive causal or direct link between the aforementioned proteins and ALS risk remains to be established, with the exception of the results reported by Chia et al. [3]. Furthermore, while our enrichment analysis revealed associations between ALS and numerous biological pathways, notably those involved in skeletal muscle function and energy metabolism, these circulating signatures do not conclusively demonstrate primary muscle pathology or cachexia, as proteins may derive from neural, hepatic or inflammatory sources. We also cannot rule out the possibility that primary alterations in muscle pathophysiology contribute to ALS pathogenesis [24]. Future studies integrating multimodal assessments are required to dissect the precise contribution of muscle tissue to the pre‐diagnostic ALS plasma proteome.

Notably, our investigation has identified a set of novel candidate proteins linked to ALS risk—specifically EGFLAM, ITGAV, THBS4 and CD248—suggesting previously unrecognized mechanisms that may underlie disease pathogenesis. EGFLAM, a protein known to mediate the alignment of photoreceptor neurons with bipolar cells and thereby ensure precise synaptic signal transmission [25], is here implicated in ALS risk for the first time, pointing to a potential role in neurodegeneration beyond the visual system. In the *SOD1* ^G93A^ mouse model of ALS, ITGAV expression was elevated in microglia and macrophages; therapeutic neutralization of ITGAV with antibodies yielded promising outcomes in a preclinical setting, ameliorating motor deficits and prolonging survival [26]. In the current investigation, plasma ITGAV levels were also identified as a potential biomarker for predicting ALS risk. THBS4, an adhesive glycoprotein that mediates cell–matrix and cell–cell interactions, plays a key role in the development and remodelling of musculoskeletal, neural and cardiovascular systems [27]. Furthermore, THBS4 has been implicated in promoting differentiation toward a pro‐inflammatory phenotype and in inducing apoptosis [28]. This broad functional involvement highlights its potential significance in the pathology of ALS. Emerging research suggests a potential association between CD248 and ALS, with one study reporting that elevated CD248 levels may correlate with a more favourable prognosis in ALS patients [29]. Its expression during the preclinical stage of the disease may further suggest a protective role in ALS pathology.

In addition to the principal analysis of general ALS, we identified exploratory evidence for plasma proteomic predictors specific to in C9‐ALS. Although the *C9orf72* HRE is a well‐established genetic cause of ALS, previous studies have revealed substantial interindividual variability, as not all carriers develop the disease—a phenomenon known as incomplete penetrance [10]. Moreover, disease risk is not strictly correlated with repeat length; rather, the presence of the expansion itself is the principal determinant of risk, with no clear linear relationship between repeat number and disease susceptibility [30]. These observations collectively suggest that additional genetic and environmental factors modulate an individual's likelihood and timing of disease onset. Our analysis indicated that, alongside NEFL, COL5A1 may also influence C9‐ALS risk relative to controls. However, likely owing to the restricted cohort size of *C9orf72* HRE carriers, only NEFL demonstrated a significant association with C9‐ALS within this subgroup. NEFL has been widely investigated as a disease modifier in C9orf72‐associated pathologies, including C9‐ALS [20]. COL5A1 supports structural integrity in skin, joints and blood vessels in classical Ehlers–Danlos syndrome [31], and elevated COL5A1 expression has been observed in various cancers, where it correlates with poor prognosis [32, 33, 34]. Interestingly, a recent study revealed elevated levels of extracellular matrix (ECM) proteins in the spinal cords of *C9orf72* knock‐in mice (GR400 and PR400 variants), with COL6A1 (Collagen Type VI Alpha 1 Chain) showing the most pronounced upregulation [35]. An ECM signature has been shown to be neuroprotective and conserved in C9‐ALS [35]. Our KEGG pathways enrichment analysis further underscored the involvement of ECM signalling, particularly ECM–receptor interactions. Consequently, elevated plasma levels of COL5A1 may promote vascular associations and inflammation‐mediated ECM disruption, thereby contributing to C9‐ALS risk. Moreover, our enrichment analysis substantiated the involvement of IL‐10‐associated immune and cytokine receptor interaction pathways in C9‐ALS pathogenesis, underscoring a critical role for specific cytokine signalling—a connection not previously established with certainty in ALS proteomic studies [3, 4, 5, 6, 7]. This interpretation is reinforced by the findings of Michael et al. [15], which indicated that T cells, especially CD4+ T cells with a propensity for releasing IL‐5 and IL‐10, contribute to C9‐ALS pathology. Taken together, these observations imply that targeting immunological or kinase‐related pathways could guide future therapeutic strategies aimed at delaying C9‐ALS onset, potentially by mitigating the downstream pathological effects driven by the *C9orf72* HRE.

Moreover, MR analysis identified a potential causal role for MYOM3 in ALS. MYOM3 is known to be involved in the pathogenesis of various myopathies [36] and has also been linked to muscle adaptation to eccentric exercise [37]. Interestingly, given epidemiological evidence linking strenuous physical activity to an elevated risk of ALS [38], dysregulation of MYOM3 or its associated pathways may provide a molecular mechanism underlying the connection between mechanical stress and motor neuron susceptibility. In contrast, for the other proteins analysed, the lack of significant associations in both the GWAS and pQTLs analysis suggests that their observed abnormalities are less likely to be genetically driven causes but rather may represent downstream consequences of the disease pathology.

Importantly, we also identified a distinct plasma protein signature capable of predicting ALS several prior to clinical onset. These protein signatures serve as a crucial factor, achieving moderate to high specificity and sensitivity and providing a basis for a biomarker‐based risk stratification strategy. Unlike the diagnostic models by Chia et al. [3], which discriminate ALS from healthy controls and other neurological diseases, our study is oriented toward prospective risk prediction. Importantly, our model accomplishes this using a markedly smaller panel of proteins. This predictive framework not only predicts ALS years in advance but also holds direct implications for early intervention trials and personalized monitoring strategies in high‐risk individuals, crucial for rapidly progressing diseases like ALS. However, the observed conservative calibration bias (slope > 1.0) underscores the inherent challenge of modelling ultra‐rare diseases like ALS.

Strengths of this study include the large sample size, prospective nature and long‐term follow‐up. In addition, Olink's ultra‐low sample requirement, high sensitivity across a broad dynamic range, excellent specificity from dual‐antibody PEA (proximity extension assay), robust standardization via NPX normalization, flexible panel design and seamless multi‐omics compatibility make it especially powerful for large‐scale cohort proteomic studies. Moreover, considering C9 HRE status enabled the discovery of more accurate protein signatures among individuals in the preclinical stage of specific ALS subgroups. Interpretation of the results should take into account certain limitations. First, the Olink proteomic measurements in UK Biobank rely on a targeted design, limited to the scope of analytes defined by the selected library panels, and notably, our study used only baseline proteomic measurements, so future studies with longitudinal proteomic profiling will be needed to validate our observations on temporal protein changes. The UKB‐PPP sample includes nonrandom strata that may introduce selection bias, but this bias is limited and can be adequately controlled by adjusting for relevant covariates, and although the mean follow‐up was 11.8 years, some control participants may develop ALS beyond the data cut‐off. Using our team's previously reported incidence estimate in Europe of 2.17 per 100 000 person‐years as a conservative lower bound [39], we estimate that < 0.5% of controls would be expected to develop ALS in the subsequent 10 years. Our primary Cox regression analysis is inherently robust to this truncation. Second, a notable limitation of this study is the lack of an independent external cohort for validation. Access to large‐scale, prospectively collected prediagnostic biospecimens remains a significant bottleneck in ALS research. Consequently, the novel protein associations identified here should be interpreted as discovery‐phase findings. Additionally, while MR provides insights into potential causal relationships, the absence of colocalization support for MYOM3 implies that horizontal pleiotropy cannot be excluded, limiting causal claims. Future collaborative efforts utilizing multi‐centre biobanks are essential to replicate these results and transition these candidates into clinically useful biomarkers. Third, our cohort contains a small subset of patients with non‐C9 HRE ALS‐causative variants (e.g., *SOD1* and *TARDBP*), which warrant careful consideration, and due to limited case numbers, C9‐ALS findings are also preliminary and should be validated in larger, targeted cohorts. Fourth, while the algorithmically defined ALS outcome reduces the risk of misclassification, we acknowledge that a degree of residual error may remain inherent to registry‐based diagnostics. Fifth, although statistical adjustments for sex were performed, the demographic skew, particularly the preponderance of females in the control group and the 93% White cohort composition, may still limit the extrapolation of our findings.

## 5 Conclusion

We discovered previously unrecognized predictive proteins associated with incident ALS and the C9‐ALS subtype. Proteins implicated in these newly identified pathological processes—including EGFLAM, ITGAV, THBS4, and CD248—may serve as reliable predictive indicators and promising therapeutic targets. Furthermore, COL5A1 shows preferential association with C9‐ALS compared to the general ALS population, indicating potential subtype‐specific pathological relevance. These findings help address critical knowledge gaps regarding early ALS pathogenesis and underscore their utility in guiding the identification of early biomarkers and the design of targeted therapeutic strategies.

## Funding

This study was supported by the National Natural Science Fund of China (grant no. 82371422 to Y.P.C.), the Science and Technology Bureau Fund of Sichuan Province (grant no. 2026NSFSC0510 to Y.P.C.), the National Key Research and Development Program of China (grant no. 2022YFC2703101 to Y.P.C.) and the 1·3·5 project for disciplines of excellence Clinical Research Fund, West China Hospital, Sichuan University (grant no. 2023HXFH032 to Y.P.C.).

## Ethics Statement

This study involved human participants and was approved by the North West Multi‐Centre Research Ethics Committee (REC reference: 21/NW/0157). Access to the UK Biobank data was granted under our approved application.

## Consent

All participants provided written informed consent prior to inclusion.

## Conflicts of Interest

The authors declare no conflicts of interest.

## Supporting information




**Figure S1:** Baseline NPX levels of 9 ALS‐associated proteins in ALS patients and controls.
**Figure S2:** Performance of a model based solely on age and sex for predicting incident all‐type ALS cases at 3, 5 and 10 years.
**Figure S3:** Internal validation of the primary predictive model for all‐type ALS cases at 3, 5 and 10 years.
**Figure S4:** Exploratory predictive model for all‐type ALS risk prediction at 3, 5 and 10 years.
**Figure S5:** Baseline NPX levels of 4 non‐C9 ALS‐associated proteins in ALS patients and controls.
**Figure S6:** Performance of a model based solely on age and sex for predicting incident non‐C9 ALS cases at 3, 5 and 10 years.
**Figure S7:** Internal validation of the primary predictive model for non‐C9 ALS cases at 3, 5 and 10 years.
**Figure S8:** Exploratory predictive model for non‐C9 ALS risk prediction at 3, 5 and 10 years.
**Figure S9:** Plasma proteins associated with incident C9‐ALS and their predictive performance.
**Figure S10:** Baseline NPX levels of 2 C9‐ALS associated proteins in ALS patients and controls.
**Figure S11:** Performance of a model based solely on age and sex for predicting incident C9 ALS cases at 3, 5 and 10 years.
**Figure S12:** Internal validation of the primary predictive model for non‐C9 ALS cases at 3, 5 and 10 years.
**Figure S13:** Exploratory predictive model for C9 ALS risk prediction at 3, 5 and 10 years.
**Figure S14:** Relationship between *C9orf72* expansion count and NEFL levels.
**Figure S15:** Violin plot showing distribution of age at onset across different ALS subgroups.
**Figure S16:** Kaplan–Meier curves showing survival probability in different ALS subgroups.
**Figure S17:** ALS‐associated plasma protein trajectories in ALS patients.
**Figure S18:** Differentially expressed proteins identified by cross‐sectional ANCOVA comparing pre‐diagnostic and symptomatic ALS stages.
**Figure S19:** Lack of significant association for candidate risk proteins in prediagnostic versus symptomatic comparisons.
**Figure S20:** GO and KEGG enrichment analyses of proteins nominally associated with C9 ALS in *C9orf72* HRE carriers.
**Figure S21:** Colocalization analysis of protein pQTL and ALS GWAS.
**Figure S22:** Schoenfeld residuals versus follow‐up time for NEFL in all‐type and non‐C9 ALS risk models.
**Figure S23:** Schoenfeld residuals versus follow‐up time for MEGF10 in all‐type and non‐C9 ALS risk models.

**Table S1:** Plasma proteins included in this study.
**Table S2:** Baseline characteristics of the internal validation cohort.
**Table S3:** Association of individual plasma proteins with incident all‐type ALS.
**Table S4:** Associations of identified plasma proteins with incident all‐type ALS in the internal validation cohort.
**Table S5:** Association of individual plasma proteins with incident non‐C9 ALS.
**Table S6:** Associations of identified plasma proteins with incident non‐C9 ALS in the internal validation cohort.
**Table S7:** Association of individual plasma proteins with incident C9 ALS.
**Table S8:** Associations of identified plasma proteins with incident C9 ALS in the internal validation cohort.
**Table S9:** Baseline characteristics of the C9orf72 HRE carrier cohort.
**Table S10:** Association of individual plasma proteins with incident C9 ALS identified in the C9orf72 HRE carrier.
**Table S11:** Associations of individual plasma proteins with age at onset of all‐type ALS.
**Table S12:** Associations of individual plasma proteins with age at onset of non‐C9 ALS.
**Table S13:** Associations of individual plasma proteins with age at onset of C9 ALS.
**Table S14:** Associations of individual plasma proteins with survival of all‐type ALS.
**Table S15:** Associations of individual plasma proteins with survival of non‐C9 ALS.
**Table S16:** Associations of individual plasma proteins with survival of C9 ALS.
**Table S17:** Mean Z‐scores of proteins in ALS patients, referenced to matched control samples.
**Table S18:** Baseline characteristics of the ALS cohort comprising patients diagnosed before and after baseline.
**Table S19:** Adjusted cross‐sectional ANCOVA for plasma proteins in pre‐diagnostic vs. symptomatic ALS stages.
**Table S20:** Gene ontology (GO) enrichment analysis for proteins associated with all‐type ALS (nominal p < 0.05).
**Table S21:** Gene ontology (GO) enrichment analysis for proteins associated with non‐C9 ALS (nominal p < 0.05).
**Table S22:** Gene ontology (GO) enrichment analysis for proteins associated with C9 ALS (nominal p < 0.05).
**Table S23:** Gene ontology (GO) enrichment analysis of C9 ALS‐associated proteins (nominal p < 0.05) identified from C9orf72 HRE carriers.
**Table S24:** Instrumental variables for 10 ALS‐associated proteins.
**Table S25:** Mendelian randomization (MR) results of plasma proteins → ALS.
**Table S26:** Results of testing for heterogeneity in MR analysis.
**Table S27:** Results of testing for pleiotropy.
**Table S28:** Colocalization results bewteen plasma proteins pQTLs and ALS GWAS.
**Table S29:** Association of identified proteins with incident ALS, stratified by age and sex subgroups.
**Table S30:** Association of identified proteins with incident ALS at 1‐, 3‐ and 5‐year lags.

**Data S1:** Supporting information.

## Data Availability

The data used in this study were obtained from the UK Biobank under a license agreement (under application 194434); access is subject to data‐use restrictions, and as such, the dataset is not publicly available.
