# Supplementary material for: Plasma Proteomic Signatures Predict Incident Amyotrophic Lateral Sclerosis: A Prospective Study Stratified by C9orf72 Expansion
Source: J Cachexia Sarcopenia Muscle. 2026 Oct 20;17(5):e70405. doi: 10.1002/jcsm.70405 (PMC13683052; doi:10.1002/jcsm.70405)
Supplement: Supplementary file 1 [file JCSM-17-e70405-s001.docx]

| **Catalog** | |
| --- | --- |
| **Figure S1** | Baseline NPX levels of 9 ALS-associated proteins in ALS patients and controls. |
| **Figure S2** | Performance of a model based solely on age and sex for predicting incident all-type ALS cases at 3, 5, and 10 years**.** |
| **Figure S3** | Internal validation of the primary predictive model for all-type ALS cases at 3, 5, and 10 years |
| **Figure S4** | Exploratory predictive model for all-type ALS risk prediction at 3, 5, and 10 years |
| **Figure S5** | Baseline NPX levels of 4 non-C9 ALS-associated proteins in ALS patients and controls. |
| **Figure S6** | Performance of a model based solely on age and sex for predicting incident non-C9 ALS cases at 3, 5, and 10 years. |
| **Figure S7** | Internal validation of the primary predictive model for non-C9 ALS cases at 3, 5, and 10 years |
| **Figure S8** | Exploratory predictive model for non-C9 ALS risk prediction at 3, 5, and 10 years |
| **Figure S9** | Plasma proteins associated with incident C9-ALS and their predictive performance |
| **Figure S10** | Baseline NPX levels of 2 C9-ALS associated proteins in ALS patients and controls |
| **Figure S11** | Performance of a model based solely on age and sex for predicting incident C9 ALS cases at 3, 5, and 10 years |
| **Figure S12** | Internal validation of the primary predictive model for non-C9 ALS cases at 3, 5, and 10 years |
| **Figure S13** | Exploratory predictive model for C9 ALS risk prediction at 3, 5, and 10 years |
| **Figure S14** | Relationship between *C9orf72* expansion count and NEFL levels. |
| **Figure S15** | Violin plot showing distribution of age at onset across different ALS subgroups. |
| **Figure S16** | Kaplan-Meier curves showing survival probability in different ALS subgroups. |
| **Figure S17** | ALS-associated plasma protein trajectories in ALS patients |
| **Figure S18** | Differentially expressed proteins identified by cross-sectional ANCOVA comparing pre-diagnostic and symptomatic ALS stages |
| **Figure S19** | Lack of significant association for candidate risk proteins in pre-diagnostic versus symptomatic comparisons |
| **Figure S20** | GO and KEGG enrichment analyses of proteins nominally associated with C9 ALS in *C9orf72* HRE carriers. |
| **Figure S21** | Colocalization analysis of protein pQTL and ALS GWAS. |
| **Figure S22** | Schoenfeld residuals versus follow-up time for NEFL in all-type and nonC9 ALS risk models |
| **Figure S23** | Schoenfeld residuals versus follow-up time for MEGF10 in all-type and nonC9 ALS risk models |

**Figure S1.** Baseline NPX levels of 9 ALS-associated proteins in ALS patients and controls.

The width of the violin reflects the density of the corresponding data. The box plots show the median (center line), the interquartile range (top and bottom edge), and the maximum and minimum (whiskers) of the NPX levels. The comparisons were conducted using Wilcoxon rank-sum test. p-values and number of cases are overlaid on the plot.

**Figure S2.** Performance of a model based solely on age and sex for predicting incident all-type ALS cases at 3, 5, and 10 years**.**

Receiver Operating Characteristic (ROC) curves evaluating the predictive accuracy of the NEFL risk model for ALS at 3 years (red), 5 years (blue), and 10 years (green). The diagonal dashed line represents the reference for no discrimination [Area Under the Curve(AUC) = 0.5]. The inset table summarizes key predictive performance metrics, including the AUC, C-index, Brier Score, Calibration slope, and the ratio of Expected to Observed events (E/O) with 95% confidence intervals (CIs) for each time point.

**Figure S3.** Internal validation of the primary predictive model for all-type ALS cases at 3, 5, and 10 years

Receiver Operating Characteristic (ROC) curves evaluating the predictive accuracy of the NEFL risk model for ALS at 3 years (red), 5 years (blue), and 10 years (green). The diagonal dashed line represents the reference for no discrimination [Area Under the Curve(AUC) = 0.5]. The inset table summarizes key predictive performance metrics, including the AUC, C-index, Brier Score, Calibration slope, and the ratio of Expected to Observed events (E/O) with 95% confidence intervals (CIs) for each time point.

**Figure S4.** Exploratory predictive model for all-type ALS risk prediction at 3, 5, and 10 years

Receiver Operating Characteristic (ROC) curves evaluating the predictive accuracy of the NEFL risk model for ALS at 3 years (red), 5 years (blue), and 10 years (green). The diagonal dashed line represents the reference for no discrimination [Area Under the Curve(AUC) = 0.5]. The inset table summarizes key predictive performance metrics, including the AUC, C-index, Brier Score, Calibration slope, and the ratio of Expected to Observed events (E/O) with 95% confidence intervals (CIs) for each time point.

**Figure S5.** Baseline NPX levels of 4 non-C9-ALS associated proteins in ALS patients and controls.

The width of the violin reflects the density of the corresponding data. The box plots show the median (center line), the interquartile range (top and bottom edge), and the maximum and minimum (whiskers) of the NPX levels. The comparisons were conducted using Wilcoxon rank-sum test. p-values and number of cases are overlaid on the plot.

**Figure S6.** Performance of a model based solely on age and sex for predicting incident non-C9-ALS cases at 3, 5, and 10 years.

Receiver Operating Characteristic (ROC) curves evaluating the predictive accuracy of the NEFL risk model for ALS at 3 years (red), 5 years (blue), and 10 years (green). The diagonal dashed line represents the reference for no discrimination [Area Under the Curve(AUC) = 0.5]. The inset table summarizes key predictive performance metrics, including the AUC, C-index, Brier Score, Calibration slope, and the ratio of Expected to Observed events (E/O) with 95% confidence intervals (CIs) for each time point.

**Figure S7.** Internal validation of the primary predictive model for non-C9 ALS cases at 3, 5, and 10 years

Receiver Operating Characteristic (ROC) curves evaluating the predictive accuracy of the NEFL risk model for ALS at 3 years (red), 5 years (blue), and 10 years (green). The diagonal dashed line represents the reference for no discrimination [Area Under the Curve(AUC) = 0.5]. The inset table summarizes key predictive performance metrics, including the AUC, C-index, Brier Score, Calibration slope, and the ratio of Expected to Observed events (E/O) with 95% confidence intervals (CIs) for each time point.

**Figure S8.** Exploratory predictive model for non-C9 ALS risk prediction at 3, 5, and 10 years

Receiver Operating Characteristic (ROC) curves evaluating the predictive accuracy of the NEFL risk model for ALS at 3 years (red), 5 years (blue), and 10 years (green). The diagonal dashed line represents the reference for no discrimination [Area Under the Curve(AUC) = 0.5]. The inset table summarizes key predictive performance metrics, including the AUC, C-index, Brier Score, Calibration slope, and the ratio of Expected to Observed events (E/O) with 95% confidence intervals (CIs) for each time point.

**Figure S9.** Plasma proteins associated with incident C9-ALS and their predictive performance.

a. Volcano plots displaying the HR (x axis) and statistical significance (−log10 of two-sided P values, y axis) for the associations of standardized protein abundance with incident C9-ALS. b. Forest plot of significantly C9-ALS-associated proteins (FDR-corrected). c. ROC using identified proteins, age, and sex to predict C9-ALS incidence at 3, 5, and 10 years.

**Figure S10.** Baseline NPX levels of 2 C9-ALS associated proteins in ALS patients and controls.

The width of the violin reflects the density of the corresponding data. The box plots show the median (center line), the interquartile range (top and bottom edge), and the maximum and minimum (whiskers) of the NPX levels. The comparisons were conducted using Wilcoxon rank-sum test. p-values and number of cases are overlaid on the plot.

**Figure S11.** Performance of a model based solely on age and sex for predicting incident C9 ALS cases at 3, 5, and 10 years.

Receiver Operating Characteristic (ROC) curves evaluating the predictive accuracy of the NEFL risk model for ALS at 3 years (red), 5 years (blue), and 10 years (green). The diagonal dashed line represents the reference for no discrimination [Area Under the Curve(AUC) = 0.5]. The inset table summarizes key predictive performance metrics, including the AUC, C-index, Brier Score, Calibration slope, and the ratio of Expected to Observed events (E/O) with 95% confidence intervals (CIs) for each time point.

**Figure S12.** Internal validation of the primary predictive model for non-C9 ALS cases at 3, 5, and 10 years

Receiver Operating Characteristic (ROC) curves evaluating the predictive accuracy of the NEFL risk model for ALS at 3 years (red), 5 years (blue), and 10 years (green). The diagonal dashed line represents the reference for no discrimination [Area Under the Curve(AUC) = 0.5]. The inset table summarizes key predictive performance metrics, including the AUC, C-index, Brier Score, Calibration slope, and the ratio of Expected to Observed events (E/O) with 95% confidence intervals (CIs) for each time point.

**Figure S13.** Exploratory predictive model for C9 ALS risk prediction at 3, 5, and 10 year

Receiver Operating Characteristic (ROC) curves evaluating the predictive accuracy of the NEFL risk model for ALS at 3 years (red), 5 years (blue), and 10 years (green). The diagonal dashed line represents the reference for no discrimination [Area Under the Curve(AUC) = 0.5]. The inset table summarizes key predictive performance metrics, including the AUC, C-index, Brier Score, Calibration slope, and the ratio of Expected to Observed events (E/O) with 95% confidence intervals (CIs) for each time point.

**Figure S14.** Relationship between *C9orf72* expansion count and NEFL levels.

A scatter plot displays individual data points, with the yellow line indicating the best-fit linear regression model. The shaded region surrounding the regression line represents the 95% confidence interval (CI) for the model. The model was adjusted for age and sex as covariates to control for potential confounding effects. Statistical significance of the association was evaluated via linear regression analysis.

**Figure S15.** Violin plot showing distribution of age at onset across different ALS subgroups.

The width of the violin reflects the density of the corresponding data. The box plots show the median (center line), the interquartile range (top and bottom edge) of the AAO. Statistical significance was determined using an unadjusted Wilcoxon rank-sum test, and an ANCOVA was used to determine adjusted means, with covariates including age, sex, ethnicity, TDI, BMI, smoking status, frequency of alcohol intake, fasting time, and sample age. P-values and group sample sizes (n) are overlaid on the plot.

**Figure S16.** Kaplan-Meier curves showing survival probability in different ALS subgroups.

Kaplan-Meier curves showing significantly shorter overall survival for C9ALS patients compared to non-C9ALS patients (log-rank test, p = 0.048).

**Figure S17.** ALS-associated plasma protein trajectories in ALS patients

**a-i:** Trajectories of ITGAV, CD248, THBS4, MYBPC1, EGFLAM, CRSP3, MYOM3, MEGF10, and COL5A1 in ALS patients. Each dot represents one patient (red: C9-ALS, blue: non-C9-ALS). Fitted curves were generated using Generalized Additive Models (GAM). The solid lines indicate model fits for C9-ALS (red) and non-C9-ALS (blue) cohorts, while the black dotted line represents the fit for the entire ALS cohort. The shaded area denotes the 95% confidence interval for the overall fit.

**Figure S18**. Differentially expressed proteins identified by cross-sectional ANCOVA comparing pre-diagnostic and symptomatic ALS stages.

Cross-sectional ANCOVA identified five differentially expressed proteins between

ALS pre-diagnostic and symptomatic ALS (a–e; FDR < 0.05): ALDH3A1 (a), GALNT5 (b), RBFOX3 (c), CHCHD6 (d), and CORO6 (e).

**Figure S19**. Lack of significant association for candidate risk proteins in pre-diagnostic versus symptomatic ALS comparisons.

None of the candidate risk proteins from the Cox PH model reached significance (FDR < 0.05) in cross-sectional ANCOVA comparing pre-diagnostic and symptomatic ALS stages. Proteins are shown in panels a–j: NEFL (a), CD248 (b), COL5A1 (c), CSRP3 (d), EGFLAM (e), ITGAV (f), MEGF10 (g), MYBPC1 (h), MYOM3 (i), and THBS4 (j).

**Figure S20.** GO and KEGG enrichment analyses of proteins nominally associated with C9 ALS in *C9orf72* HRE carriers.

**a.** Top 5 enriched GO terms for C9 ALS-associated proteins (nominal p < 0.05) identified in *C9orf72* HRE carriers. Results are displayed separately for the three GO categories: Biological Process (BP), Cellular Component (CC), and Molecular Function (MF). **b.** Top 10 enriched KEGG pathways for C9 ALS-associated proteins (nominal p < 0.05) identified in *C9orf72* HRE carriers.

**Figure S21.** Colocalization analysis of protein pQTL and ALS GWAS.

**a-j.** Locus comparison plots for (a) CD248, (b) COL5A1, (c) CSRP3, (d) EGFLAM, (e) ITGAV, (f) MEGF10, (g) MYBPC1, (h) MYOM3, (i) NEFL, and (j) THBS4 pQTLs against ALS GWAS signals. Each panel shows the -log10(P-value) for SNPs across the genomic region, colored by their linkage disequilibrium (R²) with the lead pQTL variant. The co-localization analysis was performed to assess whether genetic associations with circulating protein levels share causal variants with ALS susceptibility. The consistent scale across panels allows for direct comparison of genetic signal overlap between protein abundance and disease risk. The pQTL signals for CSRP3, MYBPC1, and MYOM3 all mapped to the same lead SNP, rs4478991.

**Figure S22.** Schoenfeld residuals versus follow-up time for NEFL in all-type and nonC9 ALS risk models

**a.**Schoenfeld residuals for NEFL plotted against follow-up time in the all-type ALS risk model. Grey points represent individual residuals at event times; the black line indicates the LOESS-smoothed trend. The horizontal dashed line at y = 0 represents the null expectation under proportional hazards. The mild downward slope suggests slight log-hazard attenuation over time (global PH test p < 0.001). **b.** Forest plot showing period-specific hazard ratios (HRs) and 95% confidence intervals (CIs) for NEFL over 0-3, 0-5, and 0-10 years in the all-type ALS risk model. Despite the mild time-varying effects, the conventional full-follow-up HR remains a valid summary, supplemented by these period-specific estimates. **c.** Schoenfeld residuals for NEFL plotted against follow-up time in the non-C9 ALS risk model. Grey points represent individual residuals at event times; the black line indicates the LOESS-smoothed trend. The horizontal dashed line at y = 0 represents the null expectation under proportional hazards. The mild downward slope suggests slight log-hazard attenuation over time (global PH test p < 0.001). **d.** Forest plot showing period-specific HRs and 95% CIs for NEFL over 0-3, 0-5, and 0-10 years in the non-C9 ALS risk model. The direction and magnitude of these estimates are broadly consistent with the conventional full-follow-up HR, confirming that mild time-varying effects do not alter the overall interpretation.

**Figure S23.** Schoenfeld residuals versus follow-up time for MEGF10 in all-type and nonC9 ALS risk models

a. Schoenfeld residuals for MEGF10 plotted against follow-up time in the all-type ALS risk model. Grey points represent individual residuals at event times; the black line indicates the LOESS-smoothed trend. The horizontal dashed line at y = 0 represents the null expectation under proportional hazards. The mild downward slope suggests slight log-hazard attenuation over time (global PH test p < 0.001). **b.** Forest plot showing period-specific hazard ratios (HRs) and 95% confidence intervals (CIs) for MEGF10 over 0-3, 0-5, and 0-10 years in the all-type ALS risk model. Despite the mild time-varying effects, the conventional full-follow-up HR remains a valid summary, supplemented by these period-specific estimates. **c.** Schoenfeld residuals for MEGF10 plotted against follow-up time in the nonC9 ALS risk model. Grey points represent individual residuals at event times; the black line indicates the LOESS-smoothed trend. The horizontal dashed line at y = 0 represents the null expectation under proportional hazards. The mild downward slope suggests slight log-hazard attenuation over time (global PH test p < 0.001). **d.** Forest plot showing period-specific HRs and 95% CIs for MEGF10 over 0-3, 0-5, and 0-10 years in the nonC9 ALS risk model. The direction and magnitude of these estimates are broadly consistent with the conventional full-follow-up HR, confirming that mild time-varying effects do not alter the overall interpretation.
