# Supplementary material for: Plasma Proteomic Signatures Predict Incident Amyotrophic Lateral Sclerosis: A Prospective Study Stratified by C9orf72 Expansion
Source: J Cachexia Sarcopenia Muscle. 2026 Oct 20;17(5):e70405. doi: 10.1002/jcsm.70405 (PMC13683052; doi:10.1002/jcsm.70405)
Supplement: Supplementary file 2 [file JCSM-17-e70405-s002.docx]

**Supplemental methods**

**Additional description of detailed methodologies, data processing workflows, and statistical analysis.**

***Blood proteomics***

Plasma proteomic profiling was performed as part of the UK Biobank Pharma Proteomics Project (UKB-PPP). From the UK Biobank cohort of over 500,000 participants, baseline plasma samples from more than 50,000 individuals were analyzed. This selection included 46,673 (86%) participants picked at random, 1,268 (2%) who took part in the COVID-19 repeat imaging study, and 6,365 (12%) selected by the 13 consortium members according to their particular research priorities. These selected samples generally represent the overall UKB population^1^. Plasma was isolated from EDTA-anticoagulated whole blood by centrifugation at 2,500 × g for 10 minutes at 4 °C, and the resulting supernatant was immediately aliquoted and stored at −80 °C until proteomic analysis. Aliquots were shipped on dry ice to the Olink Analysis Service (Sweden) for quantitative proteomic profiling using the antibody-based Olink Explore Proximity Extension Assay. Pre-analytical and analytical quality control procedures were implemented by the UKB-PPP consortium; detailed documentation is available at <https://biobank.ndph.ox.ac.uk/ukb/ukb/docs/PPP_Phase_1_QC_dataset_companion_doc.pdf>. The resultant proteomic data are reported as log2-normalized protein expression (NPX) values, enabling standardizing cross-sample analysis and comparison.

***Modelled cross-sectional temporal patterns for ALS-associated proteins***

For each identified incident ALS case, ten matched controls were selected from the reference population using the “MatchIt” package in R^2^, with matching performed on all covariates included in the Cox PH model. All protein measurements were standardized by converting to z-scores, where normalization for each individual was based on the mean and standard deviation calculated from their respective matched controls. We computed the annual average Z-score for plasma proteins deemed abnormal (absolute Z-score > 0.2) and visualized the results in a heatmap. Additionally, to model cross-sectional temporal patterns, we included participants with ALS diagnosed prior to baseline. A generalized additive model (GAM) was subsequently employed to analyze the association between ALS-related protein levels and time relative to diagnosis, encompassing both pre- and post-diagnosis periods.

***Covariates***

In line with prior research^3^, we adjusted for potential confounders, including baseline age^4^, sex^5^, ethnicity^6^, Townsend deprivation index (TDI; calculated from social class, employment status, car ownership, and housing conditions as indicators of socioeconomic status)^6^, body mass index (BMI)^7^, smoking status^8^, alcohol consumption frequency^9^, fasting duration^10^, and sample age (interval between blood collection and Olink protein measurement)^11^. For survival analysis, age at onset of ALS was additionally adjusted for alongside the aforementioned confounders.

***Cox proportional hazards models***

Cox proportional hazards (PH) models were employed to evaluate the association between plasma protein levels and incident ALS. The primary endpoint was the occurrence of ALS. Incident ALS cases were delineated as those diagnosed after the baseline visit, date (coincident with blood sample collection). Follow-up began on the date of each participant's initial assessment center visit and extended until the first occurrence of any of the following: a recorded diagnosis of ALS, death, loss to follow-up, or the study censoring date (September 2023). Protein concentrations were modeled as continuous variables (per one- Normalized Protein eXpression (NPX) increase) to enhance interpretability and reduce bias from categorization. All models were adjusted for prespecified potential confounders described above. The proportional hazards assumption was verified via Schoenfeld residual tests for all fitted Cox PH models and individual proteins^12^. Given the large UK Biobank sample size, a global Schoenfeld test p < 0.001 was taken to indicate meaningful departure from proportionality^13^. Where the global test was significant (p < 0.001) yet residuals indicated only mild log-hazard attenuation, and the protein had passed FDR < 0.05 screening in the Cox model for ALS risk, the conventional hazard ratio was reported as a summary measure with period-specific hazard ratios (0-3, 0-5, 0-10 years) provided as secondary estimates of time-varying association^13^. In contrast, survival analysis in the C9-ALS cohort were strictly exploratory owing to the limited sample size; accordingly, no further adjustments for potential non-proportionality were undertaken, even where similar patterns were observed.

***Predictive model development and performance and performance evaluation***

Time-dependent receiver operating characteristic (ROC) curves for incident ALS onset at 3, 5, and 10 years were constructed using Cox PH model. Time-dependent area under the curve (AUC) values and 95% confidence intervals were derived via 500 non-parametric bootstrap resamples of the full cohort and internal validation set. Harrell’s concordance index (C-index) was calculated to quantify global discrimination across the full follow-up period, with optimism adjustment applied to full-cohort estimates via the same bootstrap procedure. Calibration was assessed using an optimism-adjusted calibration slope (via 500 bootstrap resamples) and the expected-to-observed (E/O) event ratio. Given the rarity of ALS and the inherent challenge of estimating extreme risks in rare-disease cohorts, we pre-specified a broader acceptable range for the calibration slope (0.7–1.4) compared to common thresholds (0.9–1.1) used in high-incidence settings. An ideal slope of 1.0 indicates perfect calibration. Slopes >1.0 reflect a degree of underfitting (conservative bias), where the model's predicted risk range is insufficiently dispersed relative to observed variability—a common phenomenon in penalized Cox models with sparse events. Conversely, slopes <0.7 would indicate overfitting and an E/O ratio between 0.8 and 1.4 was considered acceptable^14-16^. Overall accuracy was measured by the integrated Brier score (IBS). The IBS provides a single summary measure of overall predictive accuracy across the entire follow-up period, quantifying the time-averaged squared discrepancy between model-predicted survival probabilities and observed event statuses, with inverse probability of censoring weighting (IPCW) applied to account for right-censored observations. Lower IBS values indicate superior overall model performance: 0 represents perfect prediction, while 0.25 corresponds to a completely uninformative model that predicts 50% survival probability for all individuals at all times^17,18^. Those identical performance metrics were applied to both sets. Furthermore, as a hypothesis-generating analysis to refine pre-symptomatic ALS risk prediction, we constructed an expanded protein signature by incorporating all additional proteins meeting a threshold (p < 0.01) in Cox PH model restricted to the derivation cohort. All analysis were performed in R. Key packages included rms, survival, pec, survminer, etc.

***Comparative proteomic analysis***

To compare proteomic profiles between pre-diagnostic ALS patients (n = 291) and symptomatic ALS before baseline (n = 45), we employed analysis of covariance (ANCOVA). Models were adjusted for baseline age, sex, ethnicity, Townsend Deprivation Index (TDI), body mass index (BMI), smoking status, alcohol consumption frequency, fasting duration, and sample age (all mentioned above). Statistical significance was defined as a false discovery rate (FDR) < 0.05.

***Enrichment analysis***

Gene Ontology (GO) term enrichment analysis for biological processes and Kyoto Encyclopedia of Genes and Genomes (KEGG) pathway enrichment analysis were performed using the ClusterProfiler package (v4.8.3), based on the list of nominally significant proteins (p < 0.05). Significant GO and KEGG terms were identified with an FDR cutoff of < 0.1.

***MR analysis***

To evaluate potential causal links between the identified proteins and ALS, a two-sample MR analysis was conducted using pQTLs and ALS GWAS data. This approach is well-established for causal inference and drug target discovery pertaining to protein traits^19-21^. The pQTLs were sourced from plasma protein GWAS summary statistics generated by the UKB-PPP of 34,557 individuals of European ancestry^1^. Instrumental variables (IVs) were selected as SNPs that reached genome-wide significance threshold (P < 5 × 10⁻⁸) and were independent based on linkage disequilibrium criteria (r² < 0.01 within a 1 Mb region). The ALS GWAS summary statistics were derived from a large-scale meta-analysis of 27,205 cases and 110,881 controls, all of European ancestry^22^. Following data harmonization, missing SNPs were replaced using proxy variants (r²≥ 0.8, if available); MR analysis were conducted using the “TwoSampleMR” package, employing the Wald ratio method for proteins with a single instrumental variable (IV) and the inverse-variance weighted (IVW) method for those with multiple IVs. Sensitivity analysis was performed to assess heterogeneity, assessed via Cochran’s Q test, and horizontal pleiotropy, evaluated using the Egger intercept test.

***Colocalization analysis***

To investigate the potential shared genetic basis between the identified plasma proteins and ALS, we conducted a Bayesian colocalization analysis using the coloc R package^23^. This analysis utilized the same ALS GWAS summary statistics^22^ and pQTLs^1^ as described for the MR analysis. Default priors were applied, with P1 and P2 set to 1×10⁻⁴ (probability of a variant being associated with one trait), and P12 set to 1×10⁻⁵ (probability of association with both traits). A colocalization signal was considered significant only if the posterior probability for a shared causal variant (PP.H4) exceeded 0.8. Moreover, we employed the *locuscomparer* package^24^ to visualize the colocalization analysis between individual protein pQTLs and the ALS GWAS.

***Sensitivity analysis***

To further evaluate the robustness of our findings, we performed sensitivity analysis on the identified proteins. First, we conducted stratified analysis by age at blood collection (<60 vs. ≥60 years) and sex (male vs. female), along with formal tests for interaction in multivariable models, to assess potential effect modification. Second, to minimize the likelihood of reverse causality, we repeated the analysis using extended lag periods of 1, 3, and 5 years after blood collection, comparing these results with those from the primary analysis without a lag period.

References:

1.	Sun, B.B. *et al.* Plasma proteomic associations with genetics and health in the UK Biobank. *Nature* **622**, 329-338 (2023).

2.	Zhang, Y. *et al.* Large-scale proteomic analyses of incident Alzheimer's disease reveal new pathophysiological insights and potential therapeutic targets. *Mol Psychiatry* **30**, 2347-2361 (2025).

3.	Gan, Y.-H. *et al.* Large-scale proteomic analyses of incident Parkinson’s disease reveal new pathophysiological insights and potential biomarkers. *Nature Aging* **5**, 642-657 (2025).

4.	Chiò, A. *et al.* ALS phenotype is influenced by age, sex, and genetics: A population-based study. *Neurology* **94**, e802-e810 (2020).

5.	Curtis, A.F. *et al.* Sex differences in the prevalence of genetic mutations in FTD and ALS: A meta-analysis. *Neurology* **89**, 1633-1642 (2017).

6.	Roberts, A.L., Johnson, N.J., Chen, J.T., Cudkowicz, M.E. & Weisskopf, M.G. Race/ethnicity, socioeconomic status, and ALS mortality in the United States. *Neurology* **87**, 2300-2308 (2016).

7.	Mariosa, D. *et al.* Body Mass Index and Amyotrophic Lateral Sclerosis: A Study of US Military Veterans. *Am J Epidemiol* **185**, 362-371 (2017).

8.	Zhan, Y. & Fang, F. Smoking and amyotrophic lateral sclerosis: A mendelian randomization study. *Ann Neurol* **85**, 482-484 (2019).

9.	Peng, B. *et al.* Role of alcohol drinking in Alzheimer’s disease, Parkinson’s disease, and amyotrophic lateral sclerosis. *International Journal of Molecular Sciences* **21**(2020).

10.	de Jong, F.A., Howlett, G.J. & Schreiber, G. Messenger RNA levels of plasma proteins following fasting. *Br J Nutr* **59**, 81-6 (1988).

11.	Enroth, S., Hallmans, G., Grankvist, K. & Gyllensten, U. Effects of Long-Term Storage Time and Original Sampling Month on Biobank Plasma Protein Concentrations. *EBioMedicine* **12**, 309-314 (2016).

12.	GRAMBSCH, P.M. & THERNEAU, T.M. Proportional hazards tests and diagnostics based on weighted residuals. *Biometrika* **81**, 515-526 (1994).

13.	Lai, Y., Zhou, H., Zhu, R., Ung, A. & Schatz, B. Population analysis of mortality risk: Predictive models from passive monitors using motion sensors for 100,000 UK Biobank participants. *PLOS Digital Health* **1**(2022).

14.	Stevens, R.J. & Poppe, K.K. Validation of clinical prediction models: what does the "calibration slope" really measure? *J Clin Epidemiol* **118**, 93-99 (2020).

15.	Martin, G.P., Riley, R.D., Collins, G.S. & Sperrin, M. Developing clinical prediction models when adhering to minimum sample size recommendations: The importance of quantifying bootstrap variability in tuning parameters and predictive performance. *Statistical Methods in Medical Research* **30**, 2545-2561 (2021).

16.	Saelmans, A. *et al.* Implementation and Updating of Clinical Prediction Models: A Systematic Review. *Mayo Clinic Proceedings: Digital Health* **3**(2025).

17.	Steyerberg, E.W. *et al.* Assessing the performance of prediction models: a framework for traditional and novel measures. *Epidemiology* **21**, 128-38 (2010).

18.	Yokota, T. *et al.* Internal validation of an 11-yr prediction model for new vertebral fractures using the vertebral bone quality score: a prospective cohort study. *JBMR Plus* **9**, ziaf155 (2025).

19.	Yin, K.F. *et al.* Systematic druggable genome-wide Mendelian randomization identifies therapeutic targets for sarcopenia. *J Cachexia Sarcopenia Muscle* **15**, 1324-1334 (2024).

20.	Duan, Q.Q. *et al.* TBK1, a prioritized drug repurposing target for amyotrophic lateral sclerosis: evidence from druggable genome Mendelian randomization and pharmacological verification in vitro. *BMC Med* **22**, 96 (2024).

21.	Su, W.M. *et al.* Systematic druggable genome-wide Mendelian randomisation identifies therapeutic targets for Alzheimer's disease. *J Neurol Neurosurg Psychiatry* (2023).

22.	van Rheenen, W. *et al.* Common and rare variant association analyses in amyotrophic lateral sclerosis identify 15 risk loci with distinct genetic architectures and neuron-specific biology. *Nat Genet* **53**, 1636-1648 (2021).

23.	Giambartolomei, C. *et al.* Bayesian Test for Colocalisation between Pairs of Genetic Association Studies Using Summary Statistics. *PLOS Genetics* **10**, e1004383 (2014).

24.	Liu, B., Gloudemans, M.J., Rao, A.S., Ingelsson, E. & Montgomery, S.B. Abundant associations with gene expression complicate GWAS follow-up. *Nat Genet* **51**, 768-769 (2019).
